# Food Safety: U.S. Rice Serves Up Arsenic

**DOI:** 10.1289/ehp.115-a296

**Published:** 2007-06

**Authors:** Carol Potera

At one point during the reign of King Cotton, farmers in the south central United States controlled boll weevils with arsenic-based pesticides, and residual arsenic still contaminates the soil. Today, rice paddies cover fields where cotton once grew, and a large market basket survey published in the 1 April 2007 issue of *Environmental Science & Technology* now shows that rice grown in this area contains, on average, 1.76 times more arsenic than rice grown in California. With rice consumption increasing steadily in the United States, high-rice diets may be of concern, says principal investigator Andrew Meharg, chair of biogeochemistry at the University of Aberdeen, United Kingdom.

Arkansas produces about half and California about 20% of the total rice grown in the United States. The rest comes from Louisiana, Mississippi, Texas, Missouri, and Florida. The total U.S. rice crop for 2004 was 6.4 million metric tons, or 1.6% of total world production, according to the USDA.

USDA data further show that U.S. rice tends to be milled and packaged close to where it is grown. About 60% of the rice grown in the United States is eaten here, and this figure has been increasing by about 2–3% a year. Rice is eaten directly or processed into breakfast cereal, rice cakes, package mixes, pet food, and beer. U.S. rice also is exported to South America, Asia, and Europe. Meharg’s team purchased 134 varieties of rice, including brown, white, organic, polished, unpolished, and instant, at grocery stores across Arkansas and California.

Meharg traced where the rice varieties originated from information on the packages and by performing a principal component analysis of selenium, cobalt, copper, and other minerals in the grain. “This elemental profile directly relates rice to soil on which it is grown,” says Meharg.

Total arsenic levels in the 107 south central rice samples averaged 0.30 μg/g, compared to an average of 0.17 μg/g in the 27 California samples. A white rice sample from Louisiana ranked highest in total arsenic (0.66 μg/g), and an organic brown rice from California ranked lowest (0.10 μg/g). Organic growing conditions, however, do not guarantee low arsenic levels, since any rice growing in arsenic-laden soil soaks up arsenic, says Meharg.

U.S. rice consumption averages about 12 grams daily, but Asian Americans average more than 115 grams daily; Hispanic and black consumers also have higher-than-average rice intakes. The U.S. EPA, which classifies inorganic arsenic as a group A human carcinogen, sets a daily limit at 10 μg/L from drinking water (the most frequent route of exposure). There is no U.S. standard for arsenic in food. However, Meharg calculated that people who eat more than 115 grams of high-arsenic rice could reach or surpass the drinking water standard.

“High-arsenic” in this instance is based on the Louisiana sample that scored highest in arsenic content, assuming that the arsenic content was 42% inorganic, as measured by Meharg in a study published in the 1 August 2005 issue of *Environmental Science & Technology*. Rice grown in Bangladesh, the world’s hot spot for arsenic poisoning, contains about 80% inorganic arsenic, and people there eat 450 grams daily.

Rice is recommended as a substitute for wheat for people with celiac disease, a condition in which the wheat protein gluten damages the intestinal lining and impairs absorption. Celiac disease afflicts 1 in 133 Americans. Gluten-free diets also are promoted for children with autistic spectrum disorders, although no clear scientific evidence supports the use of such a diet. Estimates published in the November 2001 issue of *Pediatrics* put the prevalence of autistic spectrum disorders at 6.7 children per 1,000, with 15% of these children on gluten-free diets.

The arsenic levels in U.S. rice “are possibly cause for concern,” says John Duxbury, a soil chemist at Cornell University. He completed a market basket analysis of rice purchased in upstate New York that, like Meharg’s, found high levels of arsenic in rice grown in the south central United States. But Duxbury points out that the findings are perhaps less straightforward than they may seem. In contrast to Meharg’s calculations, the U.S. rice sample with the highest arsenic in Duxbury’s unpublished analysis contained only 22% inorganic arsenic. Moreover, Duxbury’s greenhouse experiments show that farmers could significantly reduce rice arsenic levels by applying less water to the plants. Other researchers are designing rice plants that absorb less arsenic.

“Until this all gets sorted out, consumers shouldn’t be overly concerned,” Duxbury says. Nevertheless, rice fanciers might note that both Duxbury and Meharg found basmati rice imported from India and Pakistan and jasmine rice from Thailand to contain the least arsenic.

## Figures and Tables

**Figure:**
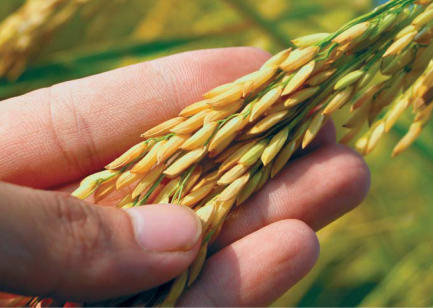
Arsenic and new rice Cotton pesticides still contaminate fields now used for food crops.

